# Microbiome: Mammalian milk microbiomes: sources of diversity, potential functions, and future research directions

**DOI:** 10.1530/RAF-23-0056

**Published:** 2024-04-12

**Authors:** Michael L Power, Carly R Muletz-Wolz, Sally L Bornbusch

**Affiliations:** 1 Center for Species Survival, Smithsonian’s National Zoo and Conservation Biology Institute, Washington, District of Columbia, USA; 2 Center for Conservation Genomics, Smithsonian’s National Zoo and Conservation Biology Institute, Washington, District of Columbia, USA; 3 Department of Nutrition Science, Smithsonian’s National Zoo and Conservation Biology Institute, Washington, District of Columbia, USA

**Keywords:** lactation, microbes, evolution, development

## Abstract

**Graphical abstract:**

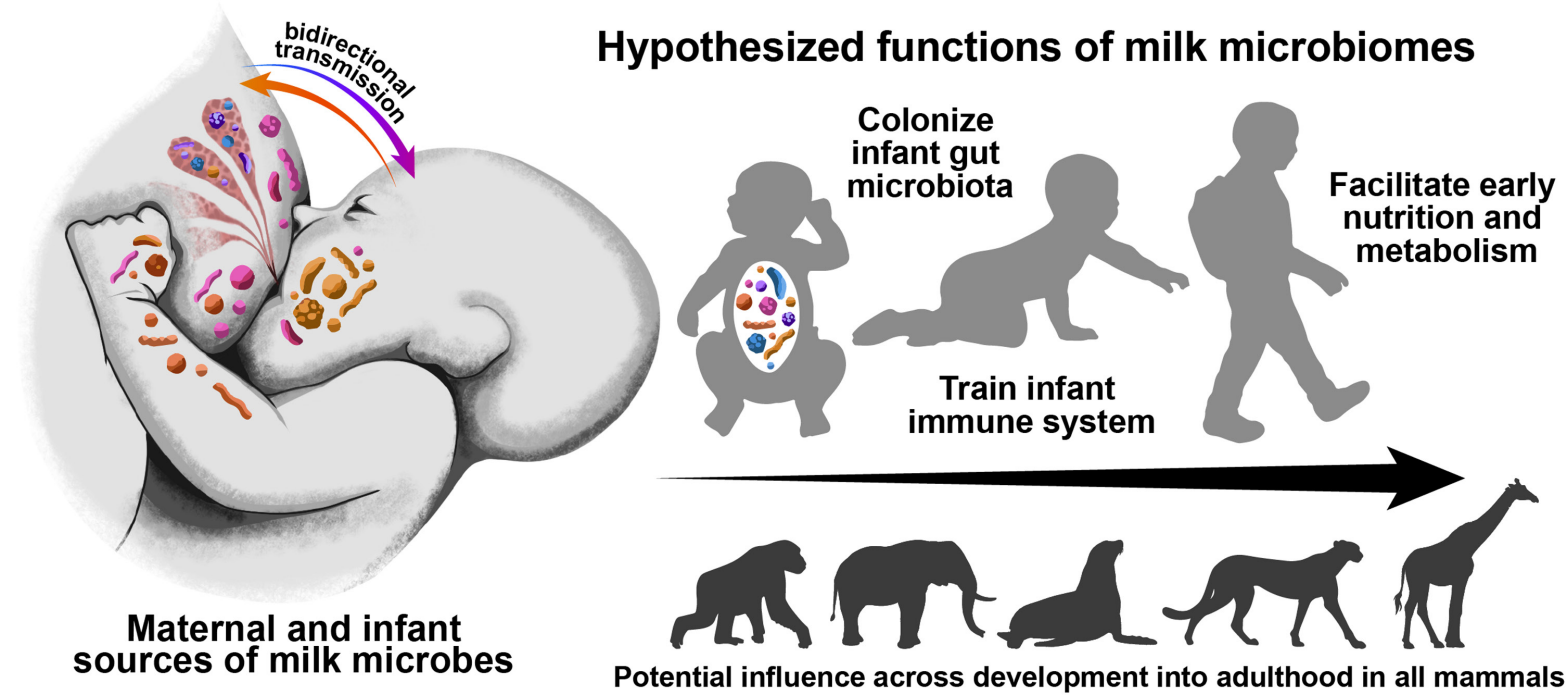

**Abstract:**

Milk is an ancient, fundamental mammalian adaptation that provides nutrition and biochemical communication to offspring. Microbiomes have been detected in milk of all species studied to date. In this review, we discuss: (a) routes by which microbes may enter milk; (b) evidence for proposed milk microbiome adaptive functions; (c) variation in milk microbiomes across mammals; and (d) future research directions, including suggestions for how to address outstanding questions on the viability and functionality of milk microbiomes. Milk microbes may be sourced from the maternal gastrointestinal tract, oral, skin, and mammary gland microbiomes and from neonatal oral and skin microbiomes. Given the variety of microbial sources, stochastic processes strongly influence milk microbiome assembly, but milk microbiomes appear to be influenced by maternal evolutionary history, diet, environment, and milk nutrients. Milk microbes have been proposed to colonize the neonatal intestinal tract and produce gene and metabolic products that influence physiology, metabolism, and immune system development. Limited epidemiological data indicate that early-life exposure to milk microbes can result in positive, long-term health outcomes. Milk microbiomes can be modified by dietary changes including providing the mother with probiotics and prebiotics. Milk replacers (i.e. infant formula) may benefit from supplementation with probiotics and prebiotics, but data are lacking on probiotics’ usefulness, and supplementation should be evidence based. Overall, milk microbiome literature outside of human and model systems is scarce. We highlight the need for mechanistic studies in model species paired with comparative studies across mammals to further our understanding of mammalian milk microbiome evolution. A broader study of milk microbiomes has the potential to inform animal care with relevance to *ex situ* endangered species.

**Lay summary:**

Milk is an ancient adaptation that supports the growth and development of mammalian neonates and infants. Beyond its fundamental nutritional function, milk influences all aspects of neonatal development, especially immune function. All kinds of milks so far studied have contained a milk microbiome. In this review, we focus on what is known about the collection of bacterial members found in milk microbiomes. Milk microbiomes include members sourced from maternal and infant microbiomes and they appear to be influenced by maternal evolutionary history, diet, milk nutrients, and environment, as well as by random chance. Once a neonate begins nursing, microbes from milk colonize their gut and produce byproducts that influence their physiology, metabolism, and immune development. Empirical data on milk microbiomes outside of humans and model systems are sparse. Greater study of milk microbiomes across mammals will expand our understanding of mammalian evolution and improve the health of animals under human care.

## Introduction

Lactation, the maternal production of milk to feed to offspring, is an ancient adaptation of the mammalian lineage. It originated in a synapsid lineage of amniotes approximately 300 million years ago, a few tens of millions of years after the divergence of the synapsids from the sauropsids (the lineage leading to reptiles and birds). The only surviving synapsid lineage is the mammalian line, suggesting that lactation may have been a fundamental adaptation for evolutionary success (Oftedal 2002, Power & Schulkin 2013).

**Figure 1:**
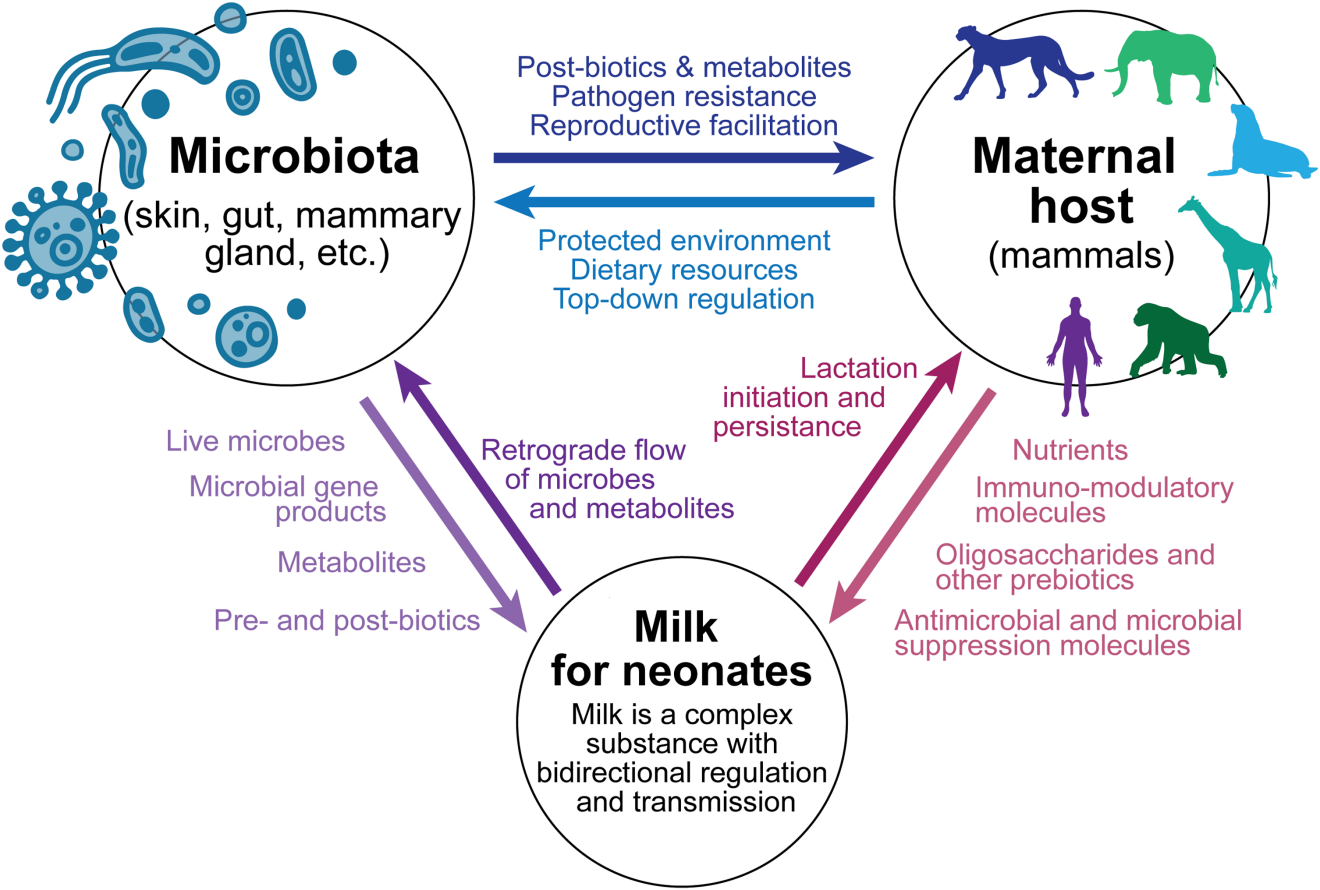
Sources and transmission of various milk components, reflecting the complexity and regulation of milk.

Milk is an incredibly complex biochemical fluid (Fig. 1) central to mammalian reproduction. The importance of milk for the nutrition of offspring is well established, as is the transfer of maternal learned immunity through immunoglobulins (e.g. IgA, IgG, IgM) and live maternal leukocytes (Power & Schulkin 2013, Bode *et al.* 2014, Goldman 2019). As technology has progressed, scientists have been able to measure a myriad of other bioactive molecules that likely affect neonatal growth and development, including hormones, growth factors, cytokines, and even microRNAs (Bartol *et al.* 2008, Power & Schulkin 2013).

As ancient as lactation and milk are, the association between microbes and vertebrates is far older. Microbes dominated the earth for over 3 billion years before mammals evolved. The entire evolution and persistence of lactation thus occurred in mammalian ancestors living in a ‘microbial world’. Milk displays signs of the evolutionary pressure exerted by microbes both in containing a myriad of antibiotic molecules and having prebiotic molecules that encourage microbial colonization of the neonatal gastrointestinal tract (Goldman 2019) (Fig. 1). Historically, milk was considered to be largely sterile. However, since the first reports of culturable and DNA-based detection of bacteria in human milk (Heikkilä & Saris 2003, Martín *et al.* 2003), all published studies have detected bacteria or bacterial DNA in at least some samples of human milk (Fernández *et al.* 2020). Even when using extreme sterile technique, research has demonstrated that there are consistent assemblages of microbes naturally occurring in milk, creating a milk microbiome (Metzger *et al.* 2018). It is likely that milk has always been associated with a microbiome, even in the initial synapsid lineage 300 million years ago when it was primarily a fluid deposited on eggs for water balance (Oftedal 2002).

We acknowledge that there remains some uncertainty about the extent and functionality of the milk microbiome. Milk samples have low microbial biomass, making them potentially susceptible to contamination and requiring particular care during analyses (Dahlberg *et al.* 2019, Douglas *et al.* 2020). Some researchers have had difficulty detecting microbial DNA in milk samples, with one study on human milk finding undetectable levels in 6 of 17 samples (Huang *et al.* 2022). Other research has suggested that a significant proportion of the microbes in milk are not viable using propidium monoazide treatment and 16S rRNA gene sequencing (PMA-seq) (Stinson *et al.* 2021 *b*).Yet PMA-seq has been shown to not always accurately quantify viable cells and in low microbial-biomass samples, such as milk, may have partial toxicity to viable cells (Wang *et al.* 2021). Multiple studies have successfully cultured bacteria from milk samples, indicating that viable bacteria are contained in milk (Heikkilä & Saris 2003, Jin *et al.* 2011, Quigley *et al.* 2013). In our own research, we have been able to extract and sequence bacterial DNA from 86% of milk samples (163/190 samples) from nine species on anthropoid primates, including humans (Muletz-Wolz *et al.* 2019), and from 86% of milk samples (107/127) from 47 species of placental mammals from all four super orders (Keady *et al.* 2023). Although there are remaining technical challenges in the detection and purification of milk microbial communities, there is a burgeoning literature supporting the existence of consistent, functional microbial communities in mammal milk that vary with host diet, phylogeny, and geographic location (Muletz-Wolz *et al.* 2019, Petrullo *et al.* 2019, Ge *et al.* 2021, Bornbusch *et al.* 2022, Keady *et al.* 2023).

In this review, we will briefly outline the evidence for a milk microbiome in a wide range of placental mammals. Compelling evidence indicates that milk hosts viral (Pitino *et al.* 2021), archaeal (Togo *et al.* 2019, Keady *et al.* 2023), bacterial (Muletz-Wolz *et al.* 2019, Bornbusch *et al.* 2022) and fungal species (Boix-Amorós *et al.* 2019). However, the vast majority of research focuses on bacterial communities, and thus the primary focus of our review will be on bacterial milk microbiomes, unless otherwise noted. We will discuss (a) the possible routes by which microbes might traverse to the mammary gland, (b) hypotheses on and evidence for potential adaptive functions of the milk microbiome, both for mother and neonate, (c) variation and influential factors in patterns of milk microbiomes in nonhuman and nonmodel animals, and (d) next research steps to test functional hypotheses and mechanisms. The majority of previous research has been focused on milk microbiomes in humans, for which there are multiple, existing reviews (e.g. Moossavi & Azad 2020, Stinson *et al.* 2021*a*). Although we pull from the more in-depth literature on human milk microbiomes, we emphasize the need to expand the study of milk microbiomes to other mammalian taxa, with particular relevance to the care of animals in *ex situ*-managed populations and in zoos. Moreover, with the increasing indirect evidence that supports hypotheses on the adaptive functions of milk microbiomes, future studies are needed to identify causal mechanisms across mammalian taxa.

## Microbiomes

There are a variety of definitions of microbiomes, all generally compatible but differing in the specifics. A widely used definition defines a microbiome as an ecological community of microorganisms (symbiotic, commensal, and pathogenic) existing within a body space or other defined environment (Lederberg & McCray 2001). Other researchers suggest an expanded ecological definition which defines a microbiome as the combination of the microbiota (the totality of live microorganisms – bacteria, archaea, fungi, algae, and protists) and the biochemical constituents of the environment in which the microbiota lives and functions (Berg *et al.* 2020). This definition includes not only the microbial community structure (microbiota) but also the gene and metabolic products produced by the microbes as well as inputs (genomic and metabolic) from the host (Fig. 1).

Most ‘microbiome’ research largely investigates the microbiota, with the bacterial members receiving the majority of scientific attention. Less attention has been paid to the environment, although that is an emerging area of research. However, the biochemical complexity of milk as an ‘environment’ likely has significant effects on milk microbiome function. Furthermore, the milk microbiome may have adaptive function in the mammary gland, in milk itself, and eventually within the gastrointestinal tract of the neonate. In this review, we will generally use the microbiome definition of Lederberg and McCray (2001) but encourage the reader to consider host inputs (maternal and neonatal) that are important for milk microbiome function (Fig. 1).

Reproductive microbiomes have traditionally been defined as microbial communities that inhabit the reproductive tract. More recently, we have expanded this to include communities outside of the reproductive tract that have demonstrable impacts on reproductive success (Comizzoli *et al.* 2021). The mammary gland is fundamentally an organ of reproduction, unique to mammals, and milk microbiomes are gaining recognition as a key reproductive microbiome that shape offspring survival and development. We include the milk microbiome within mammalian reproductive microbiomes.

## Milk microbiome formation

Unlike the majority of host-associated microbiomes (gut, skin, reproductive tract microbiomes, etc.), milk microbiomes likely have a distinct lifespan that is dictated by the production of milk and associated nursing behavior. Although there is evidence of a limited, resident mammary gland microbiota (Urbaniak *et al.* 2014*a*, Hoskinson *et al.* 2022), we hypothesize that the complexity of milk microbiomes is likely restricted to periods of lactation. This has also been proposed by Fernández *et al.* (2013) and supported by observations of lower culturable bacteria in nonlactating vs lactating individuals (Fernández *et al.* 2013, Quigley *et al.* 2013, Urbaniak *et al.* 2014*a*). However, to our knowledge, no study has examined mammary gland-associated microbiomes before, during and after lactation in the same individuals to confirm such a hypothesis. If supported, this would suggest that the microbes in milk are likely sourced from other host-associated microbiomes and the environment.

**Figure 2:**
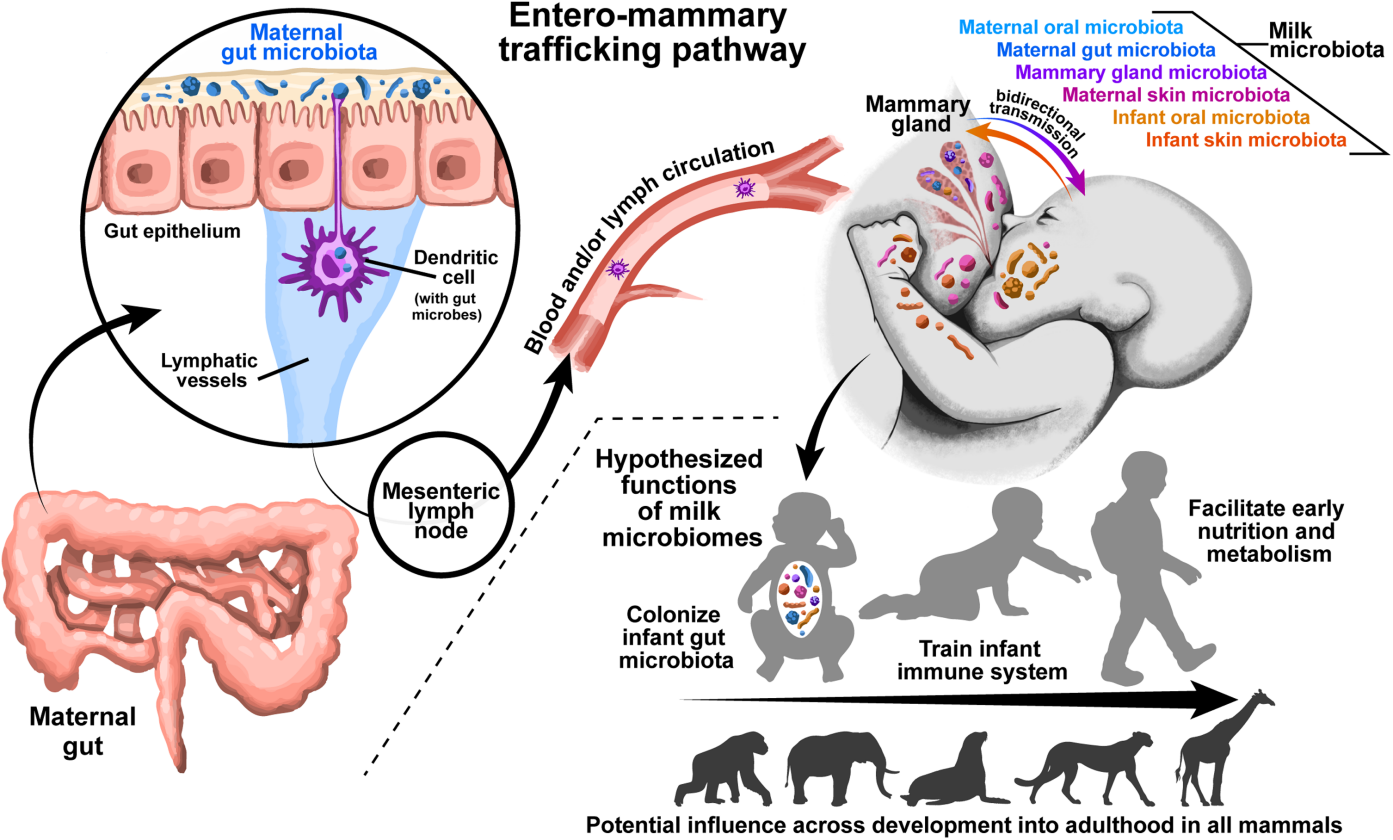
Illustration of the hypothesized entero-mammary trafficking pathway by which maternal gut microbes are transported to the mammary gland. Also illustrated are other maternal and infant sources of milk microbes and the hypothesized functions of milk microbiomes throughout development and across mammalian species.

Multiple mechanisms have been proposed for the avenues by which bacteria can colonize milk, including sources from maternal microbiomes (e.g. mammary gland, gut, skin, oral) or from offspring microbiomes (Fig. 2; as reviewed by (Fernández *et al.* 2020, Moossavi & Azad 2020)). Maternal microbiome mechanisms include: (i) the possible presence of a resident mammary gland microbiome, (ii) transfer of maternal gut-associated bacteria to the mammary gland via entero-mammary trafficking (Fig. 2), (iii) passive transport from the skin of the mother into the milk/mammary gland, and/or (4) transfer of maternal oral-associated bacteria to the mammary gland via oro-mammary translocation. Neonate-sourced microbiome mechanisms include: (i) passive transport from the skin of the infant into the mammary gland and/or (ii) retrograde flow bringing infant oral/salivary microbiomes into the mammary gland. It is important to note, that milk has a distinct bacterial community from other maternal and neonatal body sites (Biagi *et al.* 2017, Pannaraj *et al.* 2017, Williams *et al.* 2019), suggesting that milk bacteria are a conglomerate of multiple maternal and neonatal sources and that there may be physiological or microbial mechanisms that influence which microbes can persist in milk. Our recent research (Keady *et al.* 2023) also shows that stochastic ecological processes of drift and dispersal limitation play a large role in milk microbiome structure; we hypothesize that this stochasticity is a reflection of multiple sources of variable exposure to other body site microbiomes (e.g. maternal gut, maternal and neonatal oral, maternal and neonatal skin) and possibly environmental sources.

Like all other organs in the mammalian body, it is possible that there is a resident mammary gland microbiome in nonlactating mammals. The mammary gland is made up of connective tissue, fat, and tissue that contains the glands that can make milk. The primary role of the mammary gland is to produce and secrete milk to feed young offspring and is a defining feature of mammals. Fernández *et al.* (2013) originally proposed that the milk microbiome is transient: that the number of bacterial cells is not detectable in the mammary gland in nonlactating females, bacterial numbers begin to increase in the third trimester of pregnancy, sharply increase during the colostrum stage into the mature milk stage, and then sharply diminish after weaning. However, recent studies have shown that a microbiome exists in breast tissue of nonlactating human females, and a growing body of literature is examining the breast microbiome in relationship to breast cancer (e.g. Urbaniak *et al.* 2014*b*, Hoskinson *et al.* 2022). In lactating females, estimates in humans and livestock indicate that at least 1 × 10^5^ to 1 × 10^7^ colony-forming units (CFUs)/mL of bacteria are consumed daily by nursing offspring (Quigley *et al.* 2013). In human breast tissue, in the absence of lactation, estimates ranged from 75 to 2 × 10^3^ CFUs/g tissue (Urbaniak *et al.* 2014*a*), at least two orders of magnitude lower than the biomass found during lactation. This suggests that, although there may be a resident mammary gland microbiome in nonlactating females, the number and diversity of bacteria increase significantly during lactation. Thus, it is likely that mechanisms exists for increased colonization of the mammary gland by microorganisms during lactation.

Microbes may colonize milk via entero-mammary trafficking (Fig. 2), a proposed pathway by which specific bacterial taxa are trafficked from the maternal gut to the mammary gland and then to milk and the infant gut via milk consumption (Rodríguez 2014). From multiple studies in mouse models, evidence suggests that live commensal bacteria coated with immunoglobulin A reside in dendritic cells of gut-associated lymphoid tissue, particularly the mesenteric lymph node and Peyer’s patch (Macpherson & Uhr 2004, Rodríguez 2014, Treven *et al.* 2015). These microbes can be trafficked from gut-associated lymphoid tissues through the bloodstream to the mammary gland (Perez *et al.* 2007, De Andres *et al.* 2018). CD18+ cells may also be involved in trafficking gut-associated bacteria to the mammary gland (Vazquez-Torres *et al.* 1999). In a human study, lactating women given oral supplements of *Lactobacillus* had increases in the same strain in their milk, supporting the entero-mammary trafficking hypothesis (Arroyo *et al.* 2010). In a nonhuman primate model, the crab-eating macaque (*Macaca fascicularis*), it has been shown that the mammary microbiome is influenced by diet, supporting entero-mammary trafficking as one potential mechanism (Bawaneh *et al.* 2022). Additional evidence for a gut-originated milk microbiome is that many strictly anaerobic bacteria are detected in milk such as *Blautia*, *Clostridium*, *Roseburia*, *Veillonella* in humans and nonhuman primates (Muletz-Wolz *et al.* 2019, Bornbusch *et al.* 2022) and across the mammalian tree (Keady *et al.* 2023). These microbes could not originate from aerobic conditions on the skin or in the oral cavity. However, debate exists about the presence of the entero-mammary trafficking pathway (Rodríguez 2014, Petrullo *et al.* 2019). Our review of the literature and current research supports a weak influence of entero-mammary trafficking on the milk microbiome (Bawaneh *et al.* 2022, Keady *et al.* 2023), and we hypothesize that this is a result of entero-mammary trafficking being one of a series of milk bacterial inoculation pathways that may operate in nonlactating and lactating female mammals alike.

During lactation, two other pathways of milk microbiome inoculation have been proposed, which include passive transfer from maternal and neonatal skin microbiomes and active transfer from maternal (oro-mammary translocation) and neonatal (retrograde transfer) oral microbiomes. These pathways are likely common across mammalian taxa, but likely differ in contribution to seeding mammary glands based on nursing frequency and duration, integument and fur type, and neonatal oral cavity structure. It is likely that skin microbiomes contribute to the milk microbiome via maternal–offspring skin contact during breastfeeding. For instance, *Staphylococcus epidermidis* is found in both skin and milk microbiome ecosystems and shows increased abundance in the feces of human babies that are breast-fed vs formula-fed, presumably due to increased skin contact during breastfeeding (Jiménez *et al.* 2008). In addition, the passive transfer of microbes between hosts via physical contact (e.g. social and reproductive behaviors) is well established (Archie & Tung 2015, Sarkar *et al.* 2020), suggesting that a similar sharing of external microbes may occur between nursing mothers and neonates, regardless of milk production and consumption. In regards to maternal skin microbes colonizing mammary tissues and milk, bacteria have been shown to enter skin through a break or crack, usually on the nipple, and cause mastitis (Couvillion *et al.* 2023). However, to our knowledge, no studies exist on the direct transfer of commensal skin bacteria to milk or breast tissue, and it remains unclear if microbial taxa can enter healthy nipple or areola tissue. On the other hand, human studies have shown that milk microbiomes are linked to variation in infant skin and gut microbiomes, which may be related to their incidence of allergy (Gołębiewski *et al.* 2021).

The contribution of oral microbiomes – maternal and infant – to milk microbiomes is supported by evidence that they often share more similarities than are found between gut and milk microbiomes (Biagi *et al.* 2017, Drell *et al.* 2017, Moossavi *et al.* 2019, Williams *et al.* 2019). The potential of the maternal oro-mammary pathway is a new and exciting hypothesis (Moossavi & Azad 2020), based on their findings of high similarity between maternal oral and milk microbiomes (Moossavi *et al.* 2019). Much remains to be explored on this potential pathway. For the contribution of the nursing offspring’s oral microbiome to milk microbiomes, it can be difficult to determine directionality. Is it the infant oral microbiome that colonizes the milk microbiome or the milk microbiome that colonizes the infant oral microbiome? It is most plausible that the answer is both. In the infant-to-mammary gland direction, also referred to as ‘retrograde transfer’, oral-associated bacteria could enter the mammary gland via retrograde flow from the mouth or nasopharynx of the infant while suckling (Ramsay *et al.* 2004, Moossavi *et al.* 2019). Moosavi *et al.* (2019) demonstrated that the mode of breast milk feeding, either (1) nursing directly at the breast or (2) pumping and feeding from a bottle, was significantly associated with milk microbiome composition, which provides evidence for the retrograde mechanism of milk inoculation (Moossavi *et al.* 2019). However, to our knowledge, no studies exist on the direct transfer of oral bacteria to milk or mammary tissue. In the milk to infant direction, oral-associated bacteria have been isolated in precolostrum collected at the end of the first pregnancy, before any contact with the newborn, suggesting that at least some maternally sourced oral bacteria reach the infant’s mouth from the milk microbiome (Ruiz *et al.* 2019). Only specific genera are commonly shared in human milk and oral microbiomes, which include *Streptococcus* and *Staphylococcus* (Biagi *et al.* 2017, Williams *et al.* 2019), suggesting that bacterial sharing between these ecosystems is a reflection of the bacteria being able to colonize and survive in these two different ecosystems.

## Maternal and neonatal inputs to milk microbiome function

The adaptive purpose of milk is to provide neonates with the biochemical necessities for growth and development (Fig. 2). Formula-fed human babies are a testament to the fact that nutrients are the primary molecules of importance. Because they receive vital milk nutrients, they grow and develop successfully despite not receiving the myriad of nonnutritive biochemicals – and potential microbes – in breast milk. However, formula-fed neonates, human and nonhuman, often have some increased risk of pathology likely arising from developmental differences due to the lack of non-nutritional milk constituents, including those from the milk microbiome (Lyons *et al.* 2020). Some of these milk constituents interact with the microbiota in milk and may contribute to potential adaptive functions of the milk microbiome. A prime example is the oligosaccharides found in milk (Bode 2012).

All kinds of milk contain some carbohydrate. For most mammals, the primary carbohydrate is lactose, a disaccharide of glucose and galactose. All milks studied also contain other milk oligosaccharides; in humans, these can comprise 20–25% of the milk carbohydrate (Bode 2012). Milk oligosaccharides have at least four functions: (i) produce an osmotic gradient that draws intercellular water into the milk; (ii) directly interact with neonatal gut epithelial cells to enhance maturation; (iii) bind certain microbes, viruses, and other pathogens such that they travel through the neonatal gut and out in feces; and (iv) serve as prebiotics, providing presumably commensal or symbiotic microbes with a food source (Walsh *et al.* 2020). The last two functions may serve to regulate aspects of milk microbiome function, preventing colonization of the neonatal and infant gut by pathogens and encouraging and enhancing colonization by symbiotes and commensals. In addition, metabolic products created when microbes digest and utilize certain milk oligosaccharides have been shown to stimulate the development of the human infant immune system and aid in gut maturation (Walsh *et al.* 2020, Henrick *et al.* 2021). We have recently demonstrated that the sugar content in milk across 47 mammalian species is predictive of milk microbial composition and that the amount of sugar is mediated by broad host diet type (Keady *et al.* 2023). We have also shown that milk fat and protein content have links to milk microbial composition (Muletz-Wolz *et al.* 2019, Keady *et al.* 2023), highlighting that while sugar is an important nutrient with links to influencing the microbiome, other nutrient components may also play a role. Together, milk sugars and other milk nutrients directly and indirectly – through the microbiota and diet – affect neonatal health and development.

Milk is a bodily secretion that evolved to be food, making it potentially hospitable to microbes of all types. But unrestrained microbial growth within the mammary gland (e.g. mastitis) or the neonate would be maladaptive. Milk contains a wide variety of molecules whose adaptive purpose appears to be restricting growth and metabolism of microbes, ranging from bactericidal molecules such as lysozyme C (Meng *et al.* 2021) to maternal immunoglobulins that can bind pathogens (Power & Schulkin 2013). Lactoferrin binds iron in milk; iron is a limiting nutrient for many microbes. Lactoferrin has been shown to have both bacteriostatic and bactericidal activity (Sharma *et al.* 2015; Dawod *et al.* 2021) and is increased in milk during mastitis (Trajkovska *et al.* 2021). In mice, the milk complement system, comprised of maternal proteins, was recently reported to modulate neonatal gut microbiota by directly lysing certain bacteria, promoting neonatal pathogen resistance (Xu *et al.* 2024). The same study posits that the milk complement components constrain milk microbes prior to them entering the neonatal small intestine, suggesting a mechanism that modulates microbes within the mammary gland and milk (Xu *et al.* 2024). Milk thus contains biochemicals that regulate the microbiome both within the mammary gland and likely in the neonate, reflecting evolutionary pressures to nourish mammalian young, and promote symbiotic colonization of infant microbiomes, while protecting against pathogenic infection. It is feasible, even likely, that a proportion of the milk microbiome identified by DNA methods are nonviable bacterial cells and therefore may serve no active function in the mammary gland ecosystem. We suggest that future studies examine quantification methods to determine dead vs viable cells (e.g. (Wang *et al.* 2021) in milk microbiomes to understand better the functional potential of the milk microbiome.

The neonate provides the final environment for milk microbiome function. In nonruminant mammals, the pH of neonates’ stomachs differs from adults, generally being less acidic. Human babies have a postprandial stomach pH between 4 and 5 compared to an adult stomach with a pH closer to 2 (Gan *et al.* 2018). The enzyme chymosin, which breaks apart the casein micelles in milk thus forming a curd in a neonate’s stomach, functions best at that more moderate pH, while pepsin, a major stomach protease, functions best at lower pH (Gan *et al.* 2018). The lower acidity of mammalian neonate stomachs, with concomitant lower pepsin activity, would be less of a barrier to microbial transmission via ingestion.

Neonatal immune systems are immature and low functioning. The transfer of maternal learned immunity and many of the antimicrobial biochemicals in milk serve to protect the neonate in lieu of a mature immune system (Dawod *et al.* 2021). But the immature immune system also may allow colonization of the intestinal tract with maternally transmitted microbes, including those in milk. We hypothesize that one function of the milk microbiome is to educate and train the neonatal immune system to be tolerant to maternal commensal and symbiotic microbes. This would allow maternal microbes that neonates are consistently exposed to from birth through milk to be tolerated and colonize the neonatal gut. Establishing an early neonatal gut microbiome heavily influenced by maternal commensal and symbiotic microbes could serve to protect offspring from allergies (Dawod *et al.* 2021, Gołębiewski *et al.* 2021), developing atypical behavior (Kim *et al.* 2017) and metabolic disorders (Kimura *et al.* 2020), which are all pathologies linked to improper immune development. It would also help protect the neonate from pathogenic intestinal infections through competitive exclusion by benign microbes.

One possible mechanism by which milk may regulate neonate immune tolerance is through antibodies in milk, such as secretory IgA (Atyeo & Alter 2021). In humans, formula-fed infants exhibit increased gut inflammation compared to breast-fed infants (reviewed in Atyeo & Alter 2021). Evidence from human and mouse studies indicates that intestinal secretory IgA regulates both the structure and activity of commensal microbiota in the gut (Rogier *et al.* 2014). Research suggests that interactions between secretory IgA and microbial factors are one mechanism in the ‘education’ of the neonatal mucosal immune system, especially in developing tolerance to commensal microbes (Dzidic *et al.* 2018, Dawod *et al.* 2021). Similarly, milk complement has been shown to selectively cull gram-positive bacteria in the neonatal gut, while not affecting other commensal gut microbes that are presumably tolerated (Xu *et al.* 2024).

## Variation in milk microbiomes across nonhuman mammalian taxa

Variation in human milk microbiomes has been extensively studied and reviewed (Moossavi & Azad 2020, Stinson *et al.* 2021*a*); the literature on milk microbes in nonhuman animals is less robust. In this section, we briefly summarize findings on milk microbiomes in domesticated animals (agricultural and companion) and report on the limited research in wild and exotic animals. We also report on specific milk microbes found across mammalian taxa.

## Agricultural animal milk microbiomes

Given the importance of diary production worldwide, milk microbiomes have received attention for their potential impact on the dairy industry’s economic and global-health footprint. Numerous studies have reported on the milk microbial communities in healthy bovids compared to those with bovine mastitis, an infection of the mammary gland of significant economic concern for the dairy industry (Derakhshani *et al.* 2018, Hoque *et al.* 2020, Couvillion *et al.* 2023). In multiple studies, increases in the abundance of specific milk microbes, namely *Streptococcus* and *Staphylococcus* species, have been correlated with mastitis (Lima *et al.* 2017, Hoque *et al.* 2019). In many cases, these microbes are considered resident members of the bovine milk microbiome and only under certain, poorly understood conditions do these bacteria become opportunistic pathogens (Couvillion *et al.* 2023). One study identified specific lactic acid bacteria from healthy bovine mammary microbiomes as potential probiotic microbes that might prevent or treat mastitis (Bouchard *et al.* 2015). In sheep and goats, however, mastitis was associated with microbes such as *Escherichia* and *Enterococcus* that were not prevalent in healthy milk microbiomes (reviewed in Poole *et al.* 2023), suggesting that mastitis may result from infection with resident milk microbes or opportunistic pathogens that are not normally present in milks.

Beyond the impact of mastitis, multiple studies have identified milk microbiome variation in healthy dairy-producing animals, including domesticated cows, goats, sheep, buffalo, camels, and yaks (reviewed in Quigley *et al.* 2013). Across these different animals, shared and abundant bacterial genera in milks included *Lactobacillus*, *Streptococcus*, and *Staphylococcus* (Quigley *et al.* 2013). Bovine colostrum microbiomes have been reported to vary between primiparous and multiparous cows (Lima *et al.* 2017) and are posited to colonize neonatal calf gut microbiomes, with potential impacts on immune development (Hang *et al.* 2021).

Outside of dairy production, agricultural animals provide valuable systems in which to test for evidence of milk colonization pathways, such as entero-mammary trafficking. In an early study of cows, specific bacterial genera, *Ruminococcus* and *Bifidobacterium*, and members of the Peptostreptococcaceae family were found in cow milk, blood, and fecal samples, leading the authors to suggest evidence of entero-mammary trafficking (Young *et al.* 2015). In a study on domestic pigs, researchers compared the bacterial communities in maternal milk, fecal, and blood microbiomes with the aim of identifying microbes that may be transported in the bloodstream via the entero-mammary pathway (Greiner *et al.* 2022). The authors identified 117 bacterial taxa that were present in all three communities. Of these, they reported that four genera, *Clostridium sensu stricto 1*, *Mycoplasma*, *Lactobacillus*, and *Ruminococcus 2,* had abundances that varied significantly between primiparous and multiparous sows (Greiner *et al.* 2022). Overall, the current research on milk microbiomes in agricultural animals provides steppingstones for future endeavors that can have both scientific, medical, and economic benefits.

## Companion animal milk microbiomes

To our knowledge, the study of milk microbiomes in companion animals has been restricted to domestic dogs. The earliest report of milk microbes in domestic dogs was a culture-based study of milk from 44 clinically healthy dams (Kuhn *et al.* 1991). The authors report that Staphylococci were the most commonly isolated bacteria and that 67.4% of samples demonstrated moderate bacterial growth. In a more recent study of maternal (milk, vaginal, and rectal) and puppy (rectal) microbiomes, researchers report that each dam harbored a unique combination of microbes, including in milk, that contributed to shaping the puppy’s gut microbiome (Del Carro *et al.* 2022). They similarly found Staphylococci bacteria in numerous dog milk samples. There is also recent evidence that dog colostrum microbiota varies according to the type of parturition (Kajdič *et al.* 2021). Colostrum from dams that gave birth vaginally or had elective cesarean sections harbored significantly greater bacterial diversity and greater abundances of *Staphylococcus*, *Kocuria,* and *Enterococcus* compared to dams that received emergency cesarean sections (Kajdič *et al.* 2021).

Although the benefits of milk microbiomes are increasingly recognized, there are rare cases of pathogenesis in neonates apparently caused by maternal milk microbes (Zakošek Pipan *et al.* 2019). In a case study of fatal sepsis in a domestic dog puppy, researchers confirmed via bacterial whole-genome sequencing that the cause of infection was *Staphylococcus pseudintermedius* from the dam’s milk (Zakošek Pipan *et al.* 2019). Because *Staphylococcus pseudintermedius* is a common microbe found in canine milk, the authors concluded that milk microbiomes of healthy dams may rarely serve as reservoir of bacteria that can act as opportunistic pathogens in nursing offspring. In this case, it is possible that the pup was physiologically or immunologically comprised in some way and was thus unable to resist the opportunistic pathogen. Alternatively, this may have been a case of a microbe being nonharmful when in conjunction with a complete, healthy milk microbiome, but became pathogenic because it was out of balance with other members of the puppy’s gut microbiota. Cases like this raise some concern over the use of probiotics in milk replacers. Hefty doses of a few microbes may not have the appropriate regulatory or exclusion functions to keep potential pathogens from flourishing, including possibly leading a ‘benign’ microbe to overpopulate and disrupt health.

## Exotic animal milk microbiomes

Although underrepresented in the literature, studies of milk microbiomes in exotic animals provide evidence for their importance across the diverse mammalian tree. Within this subset of literature, nonhuman primates have received the most attention, with a few comparative studies examining milk microbiomes across taxonomically disparate mammals.

In nonhuman primates, multiple studies have reported variation in milk microbiome between host species and across lactation stages and have linked this variation to aspects of infant development. In an early, culture-based study of rhesus macaque milk, researchers identified 106 bacterial strains from five genera (*Bacillus, Enterococcus, Lactobacillus, Pediococcus*, and *Streptococcus*) (Jin *et al.* 2011). More recently, our study of nine nonhuman anthropoid primate species showed that overall milk community composition differed among some species, but that there was also a core set of microbes common to all nine species (Muletz-Wolz *et al.* 2019). In our subsequent study of three great ape species, a similar set of core microbes was present but varied in abundance over time (Bornbusch *et al.* 2022). We posited that the temporal variation in these core microbes may reflect the nutritional development of the infant, such as reliance on microbial contributions to milk oligosaccharide digestion. In vervet monkeys, infant gut microbiomes were found to share more microbes with maternal milk than with maternal gut microbiomes, suggesting an important role for early colonization of the infant gut (Petrullo 2022). In the same study, infant growth was correlated with the transmission and colonization of specific microbes between milk and infant guts (Petrullo *et al.* 2022), providing potential evidence for an adaptive function of milk microbiomes in determining infant growth. In our ongoing study of milk nutrients and microbiomes in four cercopithecine primates housed at three facilities, we have found that milk nutrients are largely determined by species identity whereas milk microbiomes are strongly influenced by housing facility. In our ongoing study of milk nutrients and microbiomes in four cercopithecine primates housed at three facilities, we hypothesized that milk nutrients and milk microbes are shaped by different regulatory pathways and may be under different evolutionary pressures with findings soon to be published. These results suggest that milk nutrients and milk microbes are shaped by different regulatory pathways and may be under different evolutionary pressures, with milk microbiomes showing greater population-level variation.

Broader comparative research on milk microbiomes is particularly scarce. In our recent study, we compared the milk microbiomes of 47 species spanning the placental mammalian tree, representing the most comprehensive examination of milk microbiomes to date. We found that stochastic processes (e.g. ecological drift and dispersal limitation) accounted for the majority of microbial variation (Keady *et al.* 2023). Nevertheless, deterministic factors such as host evolutionary history, diet, environment, and milk nutrients also contributed to shaping milk microbiomes (Keady *et al.* 2023). As the only known study to date that empirically examines the influence of evolutionary history on milk microbiomes, we found that host superorder (Afrotheria, Laurasiathera, Euarchontoglires, Xenarthra) accounted for ~6% of the variation in milk microbiomes. In another recent study comparing maternal milk and offspring fecal microbiomes across nine mammal species, researchers found 196 core genera across all milk microbiomes (Ge *et al.* 2021). They further reported that the contribution of milk microbiomes to offspring fecal microbiomes varied across species, with the carnivorous lions showing the greatest contribution and herbivorous goats showing the lowest. The authors theorized that this reflected the species’ digestive physiology and anatomy. The carnivore’s short, simple gut tract facilitated short gut transit time and faster passage of milk and milk microbes through the gastrointestinal tract, whereas the herbivore’s rumen fermentation and more complex digestive tract may have limited the presence of milk microbes in feces (Ge *et al.* 2021). These studies demonstrate the power of comparative research, particularly for examining evolutionary and ecological factors shaping milk microbiomes.

## Shared or ‘core’ milk microbes

With increasing study of milk microbiomes comes greater resolution into the identity of bacterial members, resulting in thousands of bacterial taxa being identified through various culturing, next-generation sequencing, and other techniques. In an assessment of existing studies on animal milk microbiomes, the genera *Streptococcus*, *Staphylococcus*, *Lactococcus*, and *Lactobacillus* were consistently identified in milks of numerous species (see supplementary table S2 in Muletz-Wolz *et al.* 2019). That assessment, however, did not examine shared taxa at strain or sequence variant level. Lactic acid bacteria are particularly abundant in most milk samples. For example, a single amplicon sequence variant identified as *Streptococcus parasanguinis* was found in two separate studies as a core milk microbe across nine primate species, including in humans (Muletz-Wolz *et al.* 2019, Bornbusch *et al.* 2022). *S. parasanguinis* is a known member of the human oral microbiomes and has been suggested as a pioneer member of infant oral cavities (Chen *et al.* 2019), suggesting increased evidence for oro-mammary trafficking. Moreover, when administered to mouse pups, *S. parasanguinis* demonstrated anti-inflammatory and immune-modulatory properties in the developing gut (Li *et al.* 2022). Other *Streptococcus* strains from agricultural milk have been reported to show adaptation to the dairy niche, including a lack of pathogenic virulence factors that are otherwise present in close taxonomic relatives (Jans *et al.* 2013, Quigley *et al.* 2013). Together, these results suggest dual selection for microbes in milk that perform specific functions (e.g. immune modulation) and pose minimal pathogenic threat to the mother and infant.

In larger comparative studies, however, there is little evidence of a core milk microbiome across the entire placental mammalian tree. Across 47 species, no strain (i.e. amplicon sequence variant) was found in greater than 40% of samples. A member of *Staphylococcus* was present in 40% of milk samples and a member of *Lactococcus* was present in all insectivores included in the study. In the same study, there was no evidence of congruence between milk microbial and host taxonomies (phylosymbiosis). Importantly, however, it is well established that taxonomically disparate bacteria can perform similar functions. We suggest that it is not necessarily a core community of microbes but a core functional capacity that may be conserved across mammals, such that specific microbes may be shared across smaller taxonomic or ecological groups (e.g. anthropoid primates or insectivores) but microbial functions may be more universally conserved across mammals. Considering the breadth of extant mammals (~6400 species), increased comparative study is needed to determine whether milk microbes and their functional capacity show similar patterns of coevolution as those found in, for example, human gut microbiomes (Suzuki *et al.* 2021).

## Knowledge gaps and future directions

Milk microbiome research is a burgeoning area of scientific, medical, and economic interest. There are, however, significant gaps in our knowledge and technical limitations that must be considered. In this section, we discuss some of these limitations and propose future directions to improve our understanding of milk microbiomes and their relevance to practical applications.

An important, yet largely unanswered, question about milk microbiomes is how maternal microbes are selected and transferred to the mammary gland. There is increasing evidence for both entero-mammary and oro-mammary trafficking as pathways that shape milk microbiomes (Drell *et al.* 2017, Williams *et al.* 2019). Across these pathways, the selectivity for specific maternal microbes and mechanisms of transport are poorly understood. There is also evidence, particularly in humans, that some mothers produce milk with very limited or undetectable microbial communities (Huang *et al.* 2022). In one study, neonates fed with ‘sterile milk’ vs ‘bacterial milk’ showed different gut microbiota, indicating that a limited or absent milk microbiota may impact the neonatal gut colonization (Huang *et al.* 2022). The reason for these minimal or absent milk communities is thus far unknown, but it has been suggested that the degree of colonization of the mammary gland and milk may vary across individuals. Addressing these gaps will require the ability to modulate maternal microbes and immune components and is likely to be best addressed in model animals. For example, genetic manipulation of the immune cells or molecules hypothesized to target and transport bacteria (e.g. dendritic cells and immunoglobins), in tandem with controlled maternal microbial communities, may shed light on the mechanisms of transport. Additionally, novel noninvasive techniques for tracking bacteria in animal hosts may help address these questions outside of model animals. For example, bacteria can be genetically engineered with acoustic reporter genes, which allows them to be located and tracked within a host’s body using noninvasive ultrasound imaging (Bourdeau *et al.* 2018), giving researchers the potential to identify microbes that are selected for the entero-mammary pathway and track their trajectory to the mammary gland. Elucidating the mechanisms behind the selection and transfer of specific maternal microbes will significantly further our understanding of vertical microbial inheritance and provide avenues for applied therapeutics (e.g. probiotics).

Although there are numerous hypothesized functions of milk microbes, rigorous testing of milk microbiome function is challenging and rare. Similarly, while studies have correlated milk microbial communities to components of neonatal health, there is a paucity of research on whether early-life exposures to milk microbiomes influence long-term development and adult health. Increasing application of techniques such as metagenomics, metatranscriptomics, and metabolomics can provide insights into the potential functional profiles of milk microbiomes. Mechanistic insights into milk microbiome function will require experimental manipulations, including varying milk microbiome structure and infant exposure to milk microbiomes, in tandem with collection of health-relevant data in developing neonates. Combining these techniques with long-term data collection from infancy to adulthood could provide more concrete evidence for the role of milk microbiomes across life-history phases.

The increased study of milk microbiomes has sparked interest in their applied value as biotherapeutics or microbial therapies. For instance, nursing mothers can take or be given probiotics to affect their milk microbiome composition (Arroyo *et al.* 2010). Although modification of maternal diet may be the most easily applied method to transform milk microbiomes to states that are supposedly more ‘beneficial’ to the offspring, much remains unknown on the connections between maternal diet, milk microbiomes, and infant health (Taylor *et al.* 2023). For human infants receiving milk formula, probiotic bacterial strains are now being included in commercial milk formulas to provide microbial ‘benefits’ (Lemoine *et al.* 2023). Some formulas additionally or separately include prebiotic molecules to promote the growth of beneficial microbes and postbiotic microbial metabolites that can promote infant health (Lemoine *et al.* 2023). In some formulas, microbes are used to ferment milk sugars, producing postbiotics and bioavailable nutrients but the live microbes are then filtered out of the milk to reduce microbial biomass in the final formula (Szajewska *et al.* 2015). Limited research suggests that when compared to traditional ‘sterile’ formulas, microbially supplemented or fermented formulas can better augment infant gut microbiomes, provide anti-inflammatory effects, and decrease disease and infection risk (Béghin *et al.* 2021, Rashidi *et al.* 2021, Lemoine *et al.* 2023). However, it is unlikely that even microbially augmented or fermented formulas could recreate the complexity of microbial interactions that occur during nursing. Further study is warranted to understand and apply probiotic supplements and dietary modifications in nursing mothers and to formulate nutritionally and microbially appropriate milk replacers for humans and nonhuman animals.

Finally, the study of milk microbiomes would benefit from an evolutionary perspective. The evolution and persistence of lactation occurred in parallel to the continuing coevolution between hosts and their microbes. Understanding the interactions between these evolutionary strategies requires a comparative approach across host taxa and could broaden our frameworks for understanding milk microbiomes. Moreover, characterizing milk microbiomes across mammalian taxa provides avenues for improving animal health and reproductive success (Comizzoli *et al.* 2021). In animals under human care, for example, milk replacers are often used for orphaned or rejected neonates. Recreating the appropriate nutritional and microbial components of that species’ milk may be a key factor in whether those infants thrive. In this manner, the study of milk microbiomes in a wide array of animals has the potential to both expand our understanding of mammalian evolution as well as improve the care and conservation of endangered animals.

## Declaration of interest

The authors declare that there is no conflict of interest that could be perceived as prejudicing the impartiality of this review.

## Funding

MLP was supported by cooperative agreement U4DMC39438: Pregnancy-Related Care Research Network from the Health Resources and Services Administrationhttp://dx.doi.org/10.13039/100000102 (HRSA) of the U.S. Department of Health and Human Serviceshttp://dx.doi.org/10.13039/100000016 (HHS). CRMW was supported by a National Science Foundationhttp://dx.doi.org/10.13039/100000001 grant (IOS-2131060). SLB was supported by a George E. Burch Postdoctoral Fellowship through the Smithsonian’s National Zoo and Conservation Biology Institute.

## Author contribution statement

MLP, CRMW, and SLB conceived, organized, and wrote this review.
